# A mink (Neovison vison) model of self-injury: Effects of CBP-CREB axis on neuronal damage and behavior

**DOI:** 10.3389/fvets.2022.975112

**Published:** 2022-11-10

**Authors:** Chunxiao Liu, Xiaolan Guo, Huazhe Si, Guangyu Li

**Affiliations:** ^1^College of Animal Science and Technology, Qingdao Agricultural University, Qingdao, China; ^2^State Key Laboratory for Molecular Biology of Special Economic Animals, Institute of Special Animal and Plant Sciences, Chinese Academy of Agricultural Sciences, Changchun, China

**Keywords:** self-injury behavior, mink, nerve damage, CBP, p-CREB

## Abstract

**Objective:**

Self-injurious behavior (SIB) is a clinically challenging problem in the general population and several clinical disorders. However, the precise molecular mechanism of SIB is still not clear. In this paper, the systematic investigation of the genesis and development of SIB is conducted based on behavioral and pathophysiology studies in mink (Neovison vison) models.

**Method:**

The night-vision video was used to observe the mink behavior, and the duration was a month. HE stain was performed to characterize the pathology change in the brain of a mink. IHC assay was performed to conduct the protein level detection of Iba-1, p-CREB, CBP, and p300 in the brain tissues. Elisa assay was used to examine the levels of NfL and NfH in serum and CSF of mink. The qRT-PCR assay was used to detect the expression of *Bcl-2, NOR1, FoxO4, c-FOS, CBP*, and *p300* in brain tissues. Western blot was used to detect the protein levels of p-CREB, CBP, and p300 in brain tissues. We also used Evans Blue as a tracer to detect whether the blood-brain barrier was impaired in the brain of mink.

**Result:**

The behavioral test, histopathological and molecular biology experiments were combined in this paper, and the results showed that CBP was related to SIB. Mechanism analysis showed that the dysregulation of CBP in brain-activated CREB signaling will result in nerve damage of the brain and SIB symptoms in minks. More importantly, the CBP-CREB interaction inhibitor might help relieve SIB and nerve damage in brain tissues.

**Conclusion:**

Our results illustrate that the induction of CBP and the activation of CREB are novel mechanisms in the genesis of SIB. This finding indicates that the CBP-CREB axis is critical for SIB and demonstrates the efficacy of the CBP-CREB interaction inhibitor in treating these behaviors.

## Introduction

Self-injurious behavior (SIB) is an intentional injury to bodily tissues without suicidal intent, and its lack of effective treatment has seriously affected global health (1). Current research on animal models of SIB has focused on drug-induced rodents and stress-stimulated rhesus monkeys. For example, Kasim et al. successfully induced self-biting behavior in experimental mice using the L-type calcium channel agonist (±)Bay K 8644 (2). Mueller et al. used the neuroexcitatory transmitter caffeine, which also caused self-biting behavior in mice (3). However, no significant physical damage was observed in this drug-stimulated self-biting model. Administration of the anxiolytic drug pemoline also causes self-injurious behavior in rats and mice by gnawing on the limbs and ventral side (4), and this model has been used to identify the neurobiological mechanisms of SIB and to evaluate potential therapeutic agents (5–13). In addition, SAPAP3 (14), Slitrk5 (15), and Shank3 (16) mutant mice exhibit SIB, resulting in tissue damage. Another important animal model of SIB, the rhesus monkey, found that migration is an important stressor that leads to increased self-biting behavior and sleeps disturbance (17). However, the underlying mechanisms of SIB are unknown and research on developing new animal models is warranted.

The American mink (Neovison vison), a semiaquatic mustelid species (18), is the most common farmed animal for fur. It has better economic importance than the silver fox, sable, marten, and skunk. However, minks are known to be susceptible to SIB, and this will hinder the development of the fur industry of minks seriously. Typical self-wounding behaviors, such as stereotypic behavior and severe tail biting, have been observed in minks. Unlike other rodent models of SIB, the pathology arises spontaneously without the need for pharmacological manipulations. Moreover, SIB in minks is always accompanied by wounds. Thus, mink may serve as an animal model to investigate the underlying biological mechanisms of SIB pathogenicity.

Cyclic adenosine monophosphate response-element-binding protein (CREB) is a significant transcriptional activator in nervous diseases (19–25). CREB comprises 341 amino acid residues, which are specifically expressed in brain tissues (26–28). The activation of CREB is mediated by phosphorylation at a specific serine residue, serine 133 (Ser133). The phosphorylation levels of CREB are markedly up-regulated in rats that are suffering the SIB (29). This phenomenon indicates that CREB signaling may play a significant role in the development of SIB. CREB binding protein (CBP), a co-activator protein, aids in assembling an active transcription complex that augments the activity of phosphorylated CREB (30, 31). Therefore, CBP may be involved in the regulation of SIB development by CREB. Although CBP is associated with a multitude of neurological disease processes (32, 33), there is no published data revealing the relationship between CBP and changes in the incidence of SIB. Here, we have made several novel observations and suggested that they are mechanistically and diagnostically indicative of SIB. Additionally, we clarified the roles of CBP in the development of SIB.

## Materials and methods

### Minks

The subjects were single-housed female American minks ranging in age from 5 to 6 months of age and maintained in standard cages. All minks were collected from the Institute of Special Economic Animal and Plant Science of the Chinese Academy of Agricultural Sciences (Jilin, China). Standard mink chow was available *ad libitum* (34, 35). The minks had wounded themselves at least once (SIB Group) with sufficient severity to require veterinary treatment, whereas the healthy minks served as the control group. Our works were approved by the Animal Care Committee of College of Animal Science and Technology, Qingdao Agricultural University (QAU). They were conducted in accordance with the QAU Guide for the Care and Use of Laboratory Animals. After behavior testing, all minks were euthanized *via* carbon dioxide, and all efforts were made to minimize suffering.

### Mice

C57BL/6 female mice were purchased from Liaoning Changsheng Biological Technology Company (Liaoning, China) and maintained in QAU animal care facilities for at least 10 days before the experiment aged 7–8 weeks were used in this work. Our works were approved by the Animal Care Committee of College of Animal Science and Technology, Qingdao Agricultural University. They were conducted in accordance with the QAU Guide for the Care and Use of Laboratory Animals. Mice were euthanized *via* carbon dioxide, and all efforts were made to minimize suffering.

### Groups

The experimental minks were divided into three independent cohorts in this study. Cohort 1: (1) Healthy minks (Control, *n* = 10), (2) Minks with SIB (SIB, *n* = 10). Cohort 2: (1) Healthy minks injected with PBS (Control + PBS, *n* = 10); (2) Healthy minks injected with CBP-CREB interaction inhibitor (hereafter called Inhibitor. Control + Inhibitor, *n* = 10); (3) Minks with SIB that injected with PBS (SIB + PBS, *n* = 15); (4) Minks with SIB were injected with Inhibitor (SIB + Inhibitor, *n* = 15). Cohort 3: (1) Healthy minks injected with PBS, after 30 min injected with Mixture (PBS + Mixture, *n* = 7). Both PBS and Mixture are at daily intervals.); (2) Healthy minks injected with Inhibitor, 30 min later, injected with Mixture (Inhibitor + Mixture, *n* = 7. Both Inhibitor and Mixture are at daily intervals.); (3) Healthy minks injected with PBS, then injected with (±) Bay K 8644 after half an hour (PBS + 8644, *n* = 7). Both PBS and (±) Bay K 8644 are at daily intervals; (4) Healthy minks injected with Inhibitor, half an hour later injected with (±) Bay K 8644 (Inhibitor + 8644, *n* = 7). Both Inhibitor and (±) Bay K 8644 are at daily intervals.

The mice were divided into four groups, detailed information in Supplementary Table 1.

### Behavioral testing

The night-vision video was purchased from PINZE (Shenzhen, China) and used to assess the mink behavior for 4 weeks (Cohort 1). Seven main categories (which include self-biting frequency, drinking frequency, sleep, sleep position, repeating wheel frequency, food intake, and defecation frequency) of behavior were recorded. The CBP-CREB interaction inhibitor was injected into minks subcutaneously for 14 days (Cohort 2). During 14 days, seven main categories were also assessed in Cohort 2. Behavioral definitions are described in detail in Supplementary Table 2.

Mice and minks (Cohort 3) in both groups (PBS + Mixture, Inhibitor + Mixture, PBS + 8644 and Inhibitor + 8644) were used to record the numbers of mice and minks that exhibited self-injuries behavior on each experimental day (The assessment was performed for 6 days). According to previous studies mice were placed in custom-made apparatus and detected with a camera to assess SIB behavior (35). Nifedipine subcutaneous injection was given when any tissue injury or bleeding occurred in mice and mink models.

### Hematoxylin-eosin staining (HE)

The brain cortex tissue was used to examine the histopathological changes of minks. The brain tissue was fixed in the 4% formalin (BBI Life Sciences Corporation, China). After embedding in paraffin, tissue sections were cut and stained with Hematoxylin and eosin (HE; Applygen Technologies Inc., China). Three micrographs per individual were performed. Sections were examined with Nano Zoomer 2.0-RS Digital Pathology (Hamamatsu, Japan).

### Immunohistochemistry (IHC)

The immunohistochemistry assay was used to analyze the protein expression of p-CREB, CBP, p300, and Iba-1. The mink brain samples were fixed in 4% paraformaldehyde and embedded in paraffin. The slides were then incubated with 3% H_2_O_2_ for 10 min to reduce non-specific staining. The treated slides were placed in a citrate buffer (pH = 6.0. Sigma-Aldrich, USA) and heated in a pressure cooker for 2 min. The slides were then incubated overnight at 4°C with four primary antibodies separately. After being washed, the slides were treated by the MaxVisionTM HRP-Polymer IHC Kit (MXB Biotechnologies, China). Then all the slides were stained with 3, 3-diaminobenzidine tetra-hydrochloride (DAB). The slides were mounted with glue for examination and capture with the Nano Zoomer 2.0-RS Digital Pathology (Hamamatsu, Japan) for comparative studies. Three micrographs per individual sample were performed. The information on the antibodies as detailed in Supplementary Table 3.

### Cerebrospinal fluid (CSF) and serum collection in mice and minks

CSF collection of minks was similar to that of mice, and this was done and described in previous references (36–38). In Cohort 1, after 4 weeks of observation, the CSF was collected immediately. In Cohort 2, the CSF was collected immediately after treating the CBP-CREB interaction inhibitor for 14 days. Then, all CSF samples were centrifuged at 15,000 × g for 1 min and stored at −80°C until their usage.

Blood samples were immediately obtained *via* the heart puncture in minks in both Cohort 1 and Cohort 2 and centrifuged at 500 × g for 15 min. Then the serum was liquated into microcentrifuge tubes and stored at −80°C for the following analysis. All blood samples were assessed macroscopically for blood contamination before the start of the experiment.

### Enzyme-linked immunosorbent assay (ELISA) for detecting neurofilament light chain (NfL) and neurofilament heavy chain (NfH) in minks and mice

CSF and Serum in Cohort 1 and Cohort 2 of minks were used to determine NfL and NfH levels. All the analyses were performed using ELISA kit. The information of the ELISA kits was described in Supplementary Table 4.

### RNA extraction and quantitative real-time polymerase chain reaction (qRT-PCR) assay

Total RNAs were extracted from mink (Cohort 1, Cohort 2 and Cohort 3) and mouse brain tissues using the TIANGEN RNA Extract kit (TIANGEN BIOTECH (BEIJING) Co., Ltd, China) according to the manufacturer's protocol. The cDNAs were synthesized from 500 ng of total RNAs using the PrimeScript RT reagent kit (Takara, Dalian, China) according to the manufacturer's protocol. The qRT-PCR assays were performed in the BioRad IQ5 Real-Time PCR System (BioRad, Hercules, CA, USA) using KAPA SYBR^®^ FAST qPCR Master Mix (KAPA, Wilmington, UK). The relative expression of RNAs was calculated using the 2^−ΔΔCT^ metho. All RNA expressions were normalized to beta-actin (β-actin). The primer sequences were described in Supplementary Table 5.

### Western blotting analysis

The minks used for experiments were euthanized, and their brain tissues were aseptically harvested. The brain tissues were subsequently treated with liquid nitrogen and lysed the brain tissue in RIPA lysis buffer (Beyotime Biotechnology, China) for 30 min. Then transferred to a centrifuge tube and centrifuged at 12,000 g for 10 min at 4°C. Then collected, the supernatant and used the BCA protein assay kit (Pierce, USA) to determine protein concentration. Total proteins were separated by 10% SDS-PAGE electrophoresis and transferred onto polyvinylidene difluoride (PVDF) membranes (Millipore Corporation, USA). The membranes were then blocked (4°C). Next, the membranes were incubated with the primary antibody overnight (4°C), followed by secondary antibodies HRP-conjugated for 1.5 h (37°C). Then using PBST washes the membranes. The protein expression of p-CREB, CBP30, and p300 were detected with the Gel Imaging System and the Quantity One Software version 4.0 (Bio-Rad, USA). Band intensity levels were normalized to β-actin (Sigma-Aldrich, USA). ImageJ analysis of protein grayscale values. The information on the antibodies as described in Supplementary Table 3.

### Evans blue analyses

At the 14 days, administered (Cohort 2) i.p. with 3% Evans Blue (EB; Sigma-Aldrich, USA) as described previously (39). Four hours after post-injection, the minks were sacrificed. The brains were harvested and fixed in 4% formalin (BBI Life Sciences Corporation, China). Frozen minks brain slabs (6 μm) are kept at cryogenic temperatures, and sections were examined in OLYMPUS Digital Pathology (OLYMPUS, Japan). ImageJ software was used as the integrated density analysis tool to measure extravascular accumulations of EB. Three micrographs per individual were performed for the photomicrographs.

### Chemicals

CBP-CREB interaction inhibitor (Catalog Number: 217505) was purchased from Merck (Germany). The inhibitor was injected into minks at a concentration of 10 mg/ml subcutaneously.

(±) Bay K 8644 agonist (Merck-Millipore, Germany) was dissolved in ethanol, then mixed with Tween 80 (Sigma) as described previously (40). Then diluted with distilled water and injected subcutaneously at a final concentration of 12 mg/ml in both mice and minks.

### Statistical analyses

All statistical analyses were performed using GraphPad Prism 5 software (Inc. 7825 Fay Avenue, Suite 230 La Jolla, CA 92037, USA). All values are expressed as mean ± SEM. Two-way ANOVA with Bonferroni post-tests and *t*-tests was used for statistical analysis. *P*-values lower than 0.05 were considered to be statistically significant.

## Result

### Behavior

**Figure 1:**
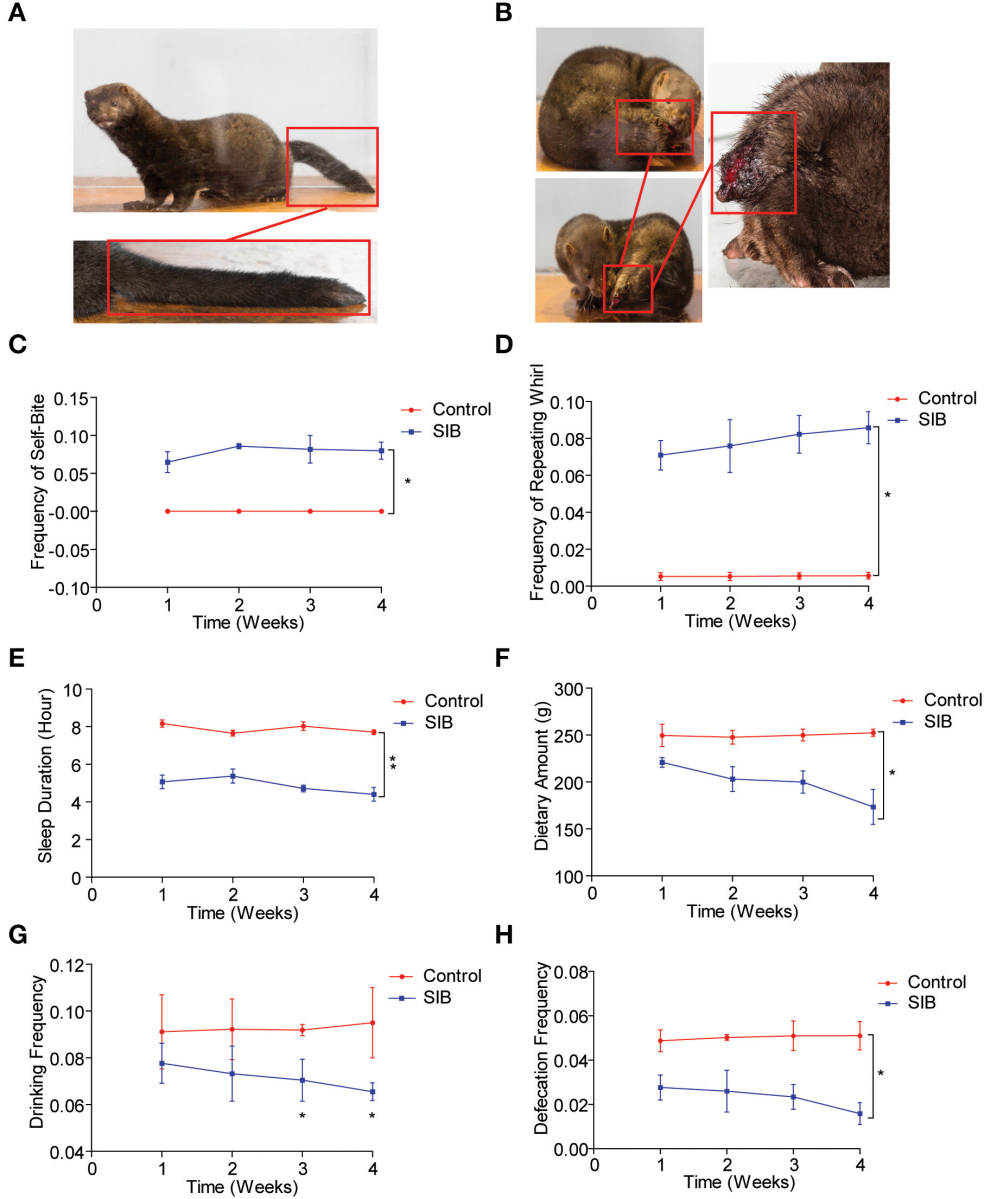
Systematic behavioral observation of minks with SIB. **(A)** Representative images of Mink in Control Group (*n* = 10). **(B)** Representative images of Mink in SIB Group (*n* = 10). **(C,D)** Frequency scores for self-biting and repeating whirl during 4 week period (data are expressed as mean ± SEM; *p < 0.05. Control, *n* = 10; SIB, *n* = 8). **(E–H)** Scores for sleeping time, dietary amount, drinking frequency, and defecation frequency during 4 weeks (data shown represent the mean ± SEM; *p < 0.05, **p < 0.01. Control, *n* = 10; SIB, *n* = 8).

An Equal number of minks (Cohort 1) was used to observe the mink behavior for 4 weeks (Control, Figure 1A; SIB, Figure 1B). Two minks were dead in the SIB group on the second and fifth day during the experiment). First, we found that the minks in the control group showed no self-biting behavior throughout the study (Figure 1C, ^*^p < 0.05). In contrast, minks with SIB exhibited severe self-biting behavior (Figure 1C, ^*^p < 0.05). Consistently, we achieved the same results of the frequency of the repeating wheel (Figure 1D, ^*^p < 0.05). Moreover, the sleep posture of minks in the SIB group exhibited a significant difference compared to the control, consistent with the previous studies (17, 41). Meanwhile, the minks with SIB indicated a significant reduction in sleeping time, dietary amount, drinking frequency and defecation frequency (Figures 1E–H, ^*^p < 0.05, ^*^^*^p < 0.01). The weights of minks were also measured, and they markedly reduced in the SIB group compared to the control (Supplementary Figure 1, ^*^^*^p < 0.01). Collectively, our results provide a systematic behavioral observation of minks with SIB.

### Minks with SIB exhibit serious nerve damage in brain

**Figure 2:**
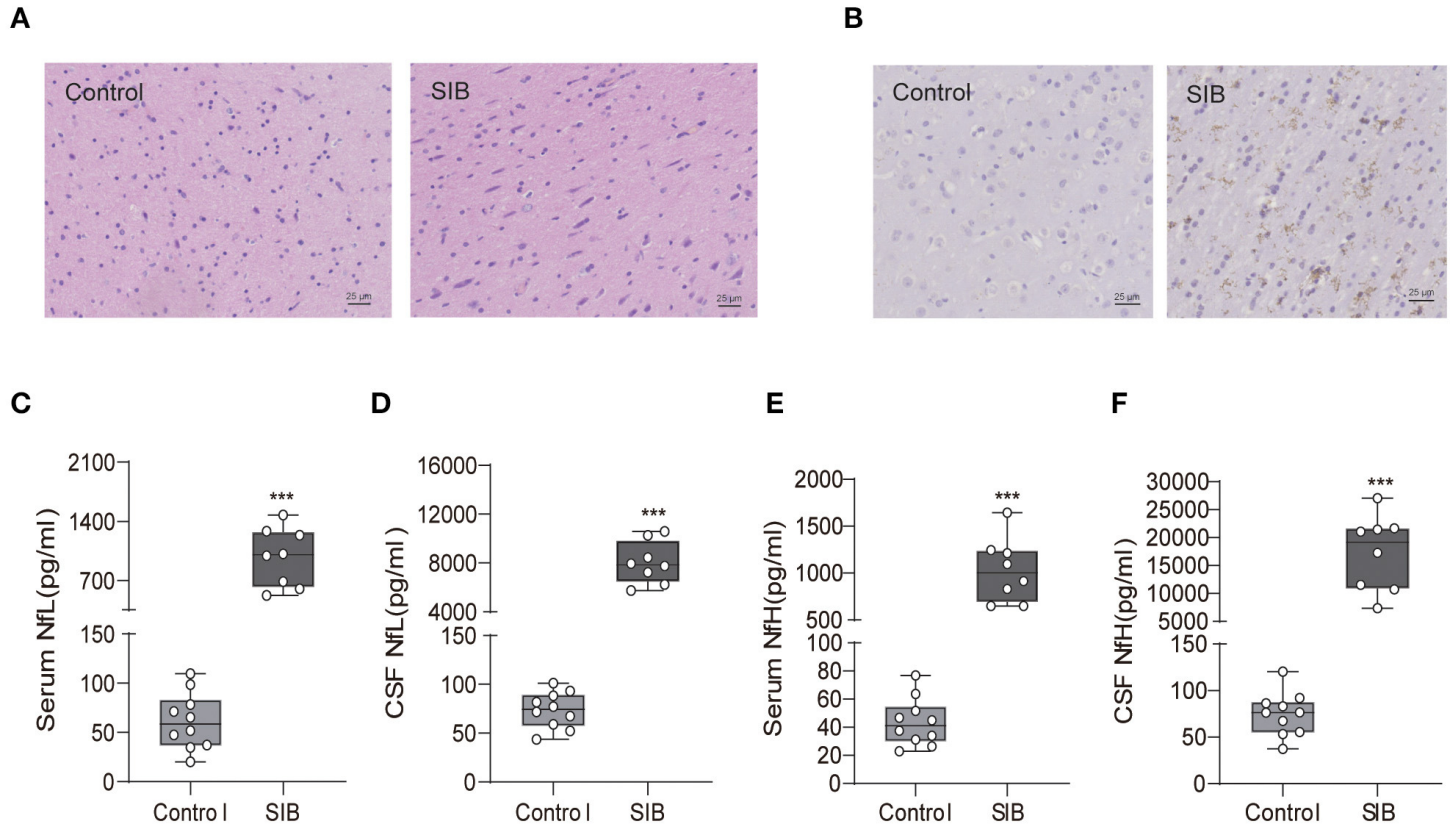
Minks with SIB exhibit serious nerve damage in brain tissues. **(A)** Microglial cells diffused hyperplasia in the brain parenchyma in SIB. Representative HE staining in the brain tissues, Scale bars, 25 μm. **(B)** Activated Iba-1 microglial cells were increased in SIB group. Representative IHC stains of Iba-1 in the brain tissues. Scale bars, 25 μm. IHC, Immunohistochemistry. Ten mink brain tissues in Control group, eight mink brain tissues in SIB group and three micrographs per individual were performed for the photomicrographs. **(C–F)** The levels of NfL and NfH in control and SIB group. Each symbol represents the serum and CSF cytokine levels (pg/ml) in one mink. Data shown represent the mean ± SEM; Control, *n* = 10; SIB, *n* = 8. ***p < 0.001.

We obtained the brain tissues from the minks of the control group and SIB group (Cohort 1) to observe the pathological change in the brain. We found that the microglial cells diffused hyperplasia in the brain parenchyma in the SIB group (Figure 2A). Control, *n* = 10; SIB, *n* = 8). Meanwhile, activated Iba-1 microglial cells were increased in SIB group (Figure 2B). Control, *n* = 10; SIB, *n* = 8). Accumulating evidence has shown that NfL and NfH can serve as reliable and easily accessible biomarkers reflecting the nervous disease progression. Thus, we examined the levels of NfL and NfH in serum and CSF of minks with SIB and controls. Indeed, we observed increased levels of NfL and NfH in the serum and CSF of SIB minks (Figures 2C–F, ^**^^*^p < 0.001). In summary, our results suggest that minks with SIB exhibit serious nerve damage in the brain.

### CBP significantly up-regulated in the brain tissues of minks with SIB

**Figure 3:**
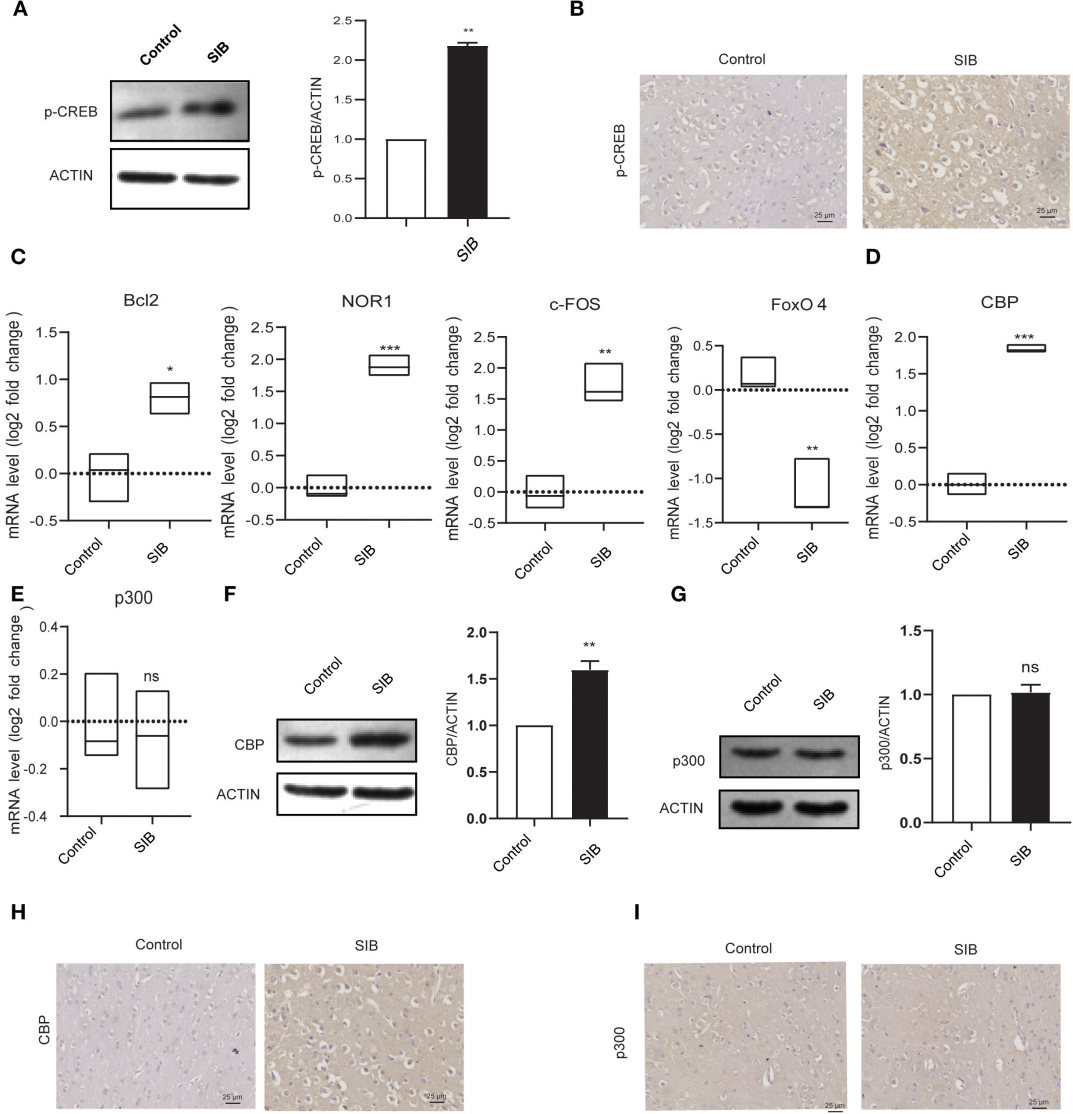
CBP and p-CREB are significantly increased in Mink brain. **(A)** The protein levels of p-CREB in brain tissues, assayed by western blot. **(B)** Representative IHC stains of p-CREB in the brain tissues. Scale bars, 25 μm. Three micrographs per individual were performed for the photomicrographs. **(C)** The mRNA levels of *Bcl2, NOR1, FoxO4*, and *c-FOS* in brain tissues, assayed by qRT-PCR. **(D)** The mRNA levels of *CBP* in brain tissues, assayed by qRT-PCR. **(E)** The mRNA levels of *p300* in brain tissues, assayed by qRT-PCR. **(F)** The protein levels of CBP in brain tissues, assayed by western blot. **(G)** The protein levels of p300 in brain tissues, assayed by western blot. **(H)** Representative IHC stains of CBP in the brain tissues. Scale bars, 25 μm. Three micrographs per individual were performed for the photomicrographs. **(I)** Representative IHC stains of p300 in the brain tissues. Scale bars, 25 μm. Three micrographs per individual were performed for the photomicrographs. Data shown represent the mean ± SEM; Control, *n* = 10; SIB, *n* = 8. *p < 0.05, **p < 0.01, ***p < 0.001, n.s., no significance.

Next, we investigated the precise molecular mechanism of SIB. Previous studies showed that the phosphorylation levels of CREB were markedly up-regulated in rats with SIB (29), suggesting CREB signaling may play a significant role in the genesis and development of SIB. Indeed, we also found that phosphorylation levels of CREB were significantly increased in the brain tissues of minks with SIB compared to controls (Figures 3A,B). In addition, we investigated whether the CREB signaling is significantly enhanced in brain tissues of minks with SIB by detecting the direct target genes of *CREB, Bcl2, NOR1*, and *c-FOS*, which *CREB* directly transactivated, were significantly increased in brain tissues of minks with SIB (Figure 3C, ^*^^*^p < 0.01, ^**^^*^p < 0.001). In contrast, *FoxO4*, which CREB directly suppresses, was reduced in brain tissues of minks with SIB (Figure 3C, ^**^^*^p < 0.001). Collectively, these results indicate that CREB signaling is significantly enhanced in the brain of minks with SIB and may play crucial roles in the genesis and development of SIB.

CREB-binding protein (CBP) and its paralog p300, which were originally identified as the transcriptional cofactors of CREB, may significantly enhance the transcriptional activity of phosphorylated CREB (30, 31). Given that CREB signaling is significantly activated in the brain of minks with SIB, we hypothesized that CBP and p300 might involve in this process. To test the hypothesis, we first investigated whether the mRNA levels of *CBP* and *p300* were also increased in the brain tissues of minks with SIB compared to controls (Cohort 1). Indeed, the mRNA levels of *CBP* exhibited a significant increase in the brain tissues of the SIB group (Figure 3D, ^**^^*^p < 0.001). However, the mRNA levels of *p300* showed no change (Figure 3E, n.s.), indicating that p300 may not affect the CREB signaling in the brain tissues of minks. Consistently, the protein levels of CBP were up-regulated in the brain tissues of the SIB group, but not p300 (Figures 3F,G). Furthermore, immunohistochemical results also confirmed that the level of CBP was significantly higher in the brain tissue of the SIB group, but there was no significant difference in p300 (Figures 3H,I). In summary, these results indicate that CBP is significantly increased in brain tissues of minks with SIB and thus activates CREB signaling, resulting in the genesis and development of SIB.

### CBP-CREB interaction inhibitor significantly relieves SIB symptoms

**Figure 4:**
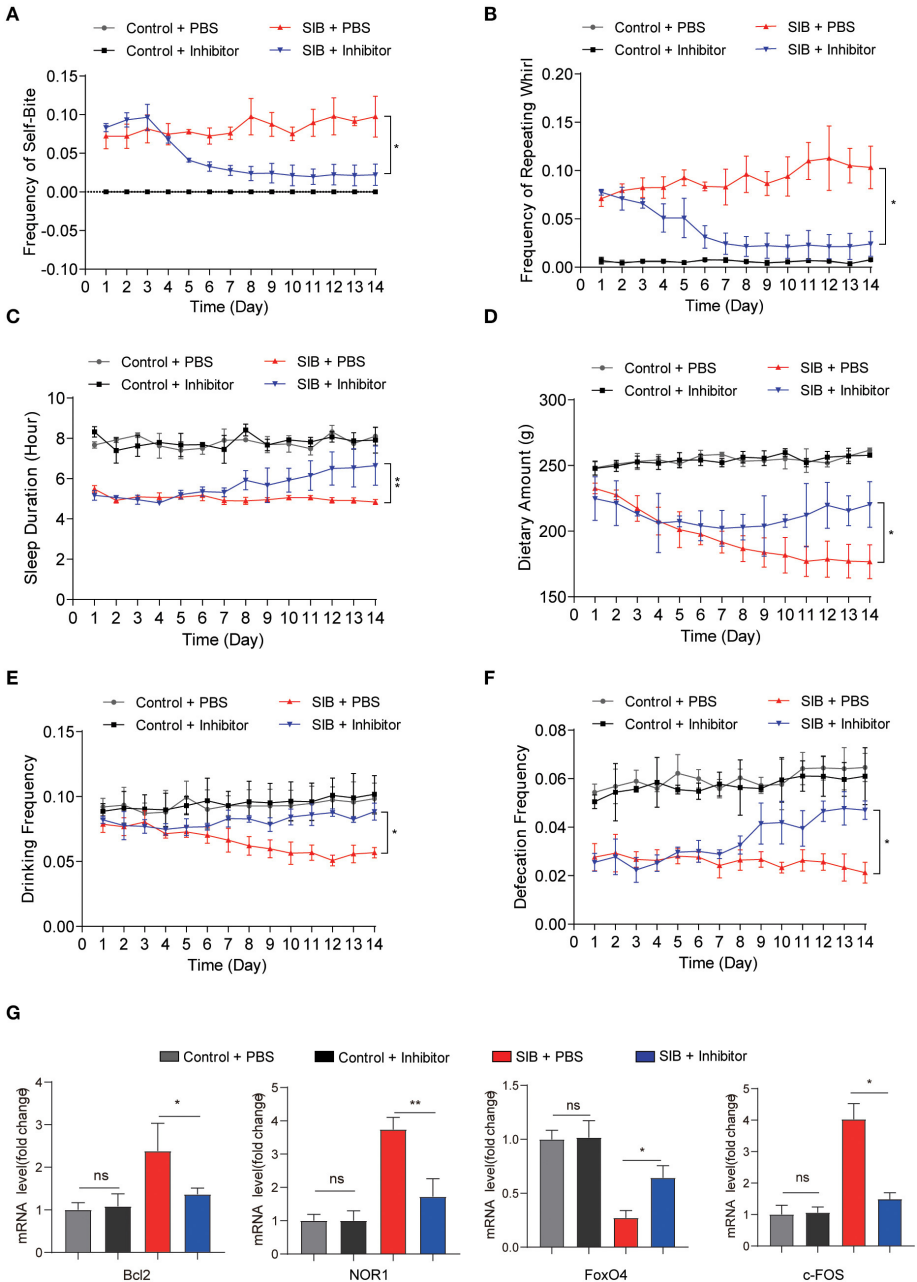
CBP inhibitor significantly relieves SIB symptoms. **(A,B)** Frequency scores for self-biting and repeating whirl during 14 days. **(C–F)** Frequency scores for sleeping time, dietary amount, drinking frequency and defecation frequency during 14 days. **(G)** The mRNA levels of *Bcl2, NOR1, FoxO4*, and *c-FOS* in brain tissues after 14 days treatment with inhibitor or PBS, assayed by qRT-PCR. Data shown represent the mean ± SEM; Control + PBS, *n* = 10; Control + inhibitor, *n* = 10; SIB + PBS, *n* = 13; SIB + inhibitor, *n* = 14. *p < 0.05, **p < 0.01, n.s., no significance.

To validate the effect of CBP and CREB signaling on the genesis and development of SIB, we used a CBP-CREB interaction inhibitor (hereafter called Inhibitor) to inhibit the interaction between CBP and CREB *in vivo* (Cohort 2). Compared to the SIB + PBS group, the minks in the SIB + Inhibitor group exhibited a significant reduction in the frequency of self-biting and repeating wheel (Figures 4A,B, ^*^p < 0.05. Two minks in the SIB + PBS group were dead at the second day of the experiment and one mink in SIB + Inhibitor was dead at the third day of the experiment.). Moreover, sustained administration of Inhibitor improved duration of the sleep of minks with SIB (Figure 4C, ^*^^*^p < 0.01) and increased dietary amount (Figure 4D, ^*^p < 0.05), drinking frequency (Figure 4E, ^*^p < 0.05) and defecation frequency (Figure 4F, ^*^p < 0.05). In addition, the Inhibitor-treated minks with SIB gained weight over 14 days, but not the PBS-treated SIB group (Supplementary Figure 2, ^*^p < 0.05). The Inhibitor treatment significantly promoted wound healing (10 of the 14 minks, 71.4%) until the end of the 14-day monitoring period (Supplementary Figure 3). These results showed that sustained administration of Inhibitor gradually relieved the self-injury behavior.

Next, we examined whether the CREB signaling was inhibited after consecutive Inhibitor injections. To this end, we used qRT-PCR assay to detect the target genes of *CREB*. We found that Inhibitor treatment efficiently decreased the expression of *Bcl2, NOR1*, and *c-FOS* (Figure 4G, ^*^p < 0.05, ^*^^*^p < 0.01), while the expression of *FoxO4* was increased upon the Inhibitor treatment (Figure 4G, ^*^p < 0.05). Importantly, we also found that the expression of *CBP* was significantly increased in the SIB mice and mink models induced by (±) Bay K 8644 agonist (Supplementary Figure 4, ^*^^*^p < 0.01). Meanwhile, the Inhibitor also relieved the self-injury behavior in the induced SIB mice and mink models (Supplementary Tables 6, 7). These results suggest that the CBP-CREB interaction inhibitor significantly suppresses CREB signaling and thereby relieves the self-injury behavior.

### Pathological change after treatment with CBP-CREB interaction inhibitor

**Figure 5:**
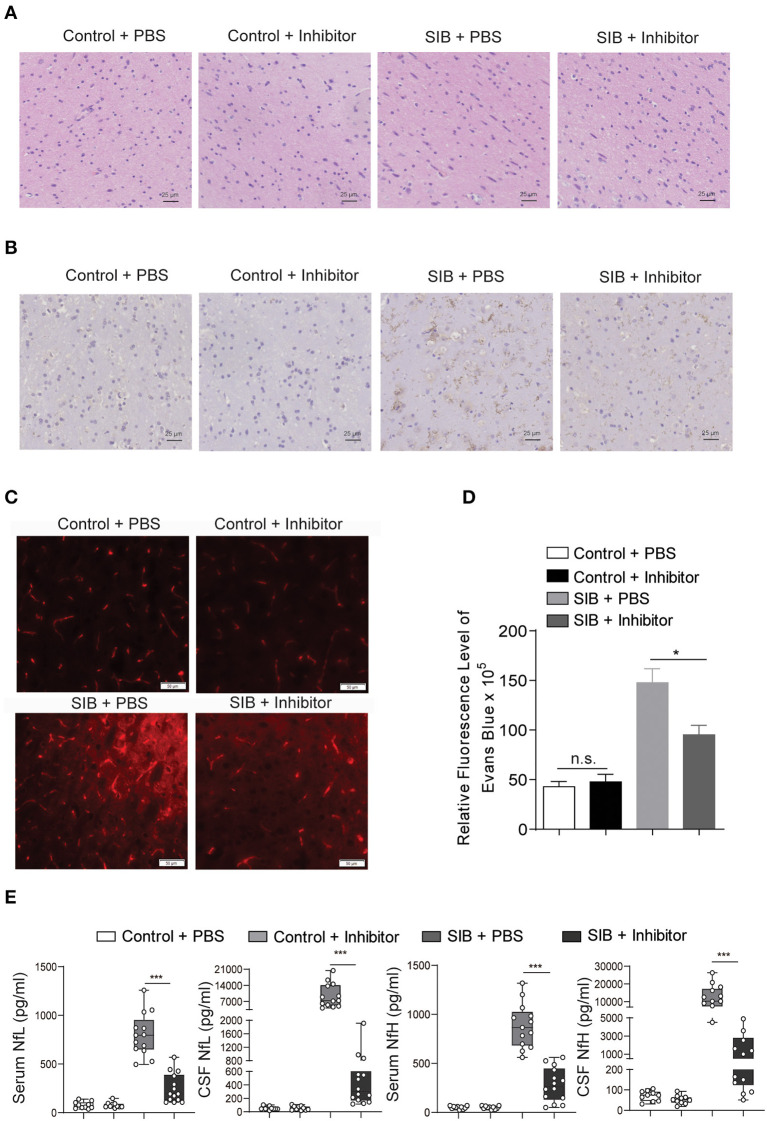
CBP inhibitor markedly relieves the nerve damage in brain tissues. **(A)** The hyperplasia of microglial cells was relieved by inhibitor. Representative HE staining in the brain tissues. Scale bars, 25 μm. Three micrographs per individual were performed for the photomicrographs. **(B)** Activated Iba-1 microglial cells were decreased in SIB + CBP30 group. Representative IHC stains of Iba-1 in the brain tissues. Scale bars, 25 μm. Three micrographs per individual were performed for the photomicrographs. **(C)** Representative images of Evans Blue levels. Scale bars, 50 μm. Three micrographs per individual were performed for the photomicrographs. **(D)** Quantifications of Evans Blue levels. **(E)** The levels of NfL and NfH in both four groups. Each symbol represents the serum and CSF cytokine levels (pg/ml) in one mink. Data shown represent the mean ± SEM; Control + PBS, *n* = 10; Control + inhibitor, *n* = 10; SIB + PBS, *n* = 13; SIB + inhibitor, *n* = 14. *p < 0.05, ***p < 0.001, n.s., no significance.

Given that the CBP-CREB interaction inhibitor may relieve the self-injury behavior, we hypothesized that it might rescue the nerve damage in brain tissues of minks with SIB. Indeed, the hyperplasia of microglial cells was relieved by an Inhibitor in the brain parenchyma of minks with SIB (Figure 5A). Compared to the SIB + PBS group, activated Iba-1 microglial cells were decreased in brain tissues of the SIB + Inhibitor group (Figure 5B). We used Evans Blue as a tracer to detect whether the blood-brain barrier was impaired in the brain of mink with SIB. The results showed that the blood-brain barrier was impaired in the brain of mink with SIB, and Inhibitor may relieve the injury (Figures 5C,D, ^*^p < 0.05). In addition, the levels of NfL and NfH in the serum and CSF of minks with SIB were also decreased upon Inhibitor treatment (Figure 5E, ^**^^*^p < 0.001). These results indicate that the CBP-CREB interaction inhibitor markedly relieved the nerve damage in brain tissues, and offers the intriguing possibility that the CBP-CREB axis may serve as novel and accessible markers of SIB disease progression.

## Discussion

In the present study, we provided a systematic behavioral analysis of SIB in minks for the first time. Meanwhile, we observed that minks with SIB exhibited serious nerve damage in brain tissues. Mechanistically, CBP was significantly increased and activated CREB signaling in the brain tissues of the SIB minks, indicating the significant role of the CBP-CREB axis in the elicitation of SIB. Furthermore, an important finding was that inhibitors of CBP could improve behavioral and physiological disorders of minks with SIB *in vivo*, suggesting that CBP was a critical molecular for SIB and it might be potentially an effective target for SIB therapy.

SIB occurs in several neurological and neuropsychiatric conditions. However, the neuropathology of SIB has not been systematically explored. Because of the ethical difficulties in carrying out the study in human subjects, experimental animal models were used to investigate SIB. Several animal models of SIB have been described (17, 29). For example, Matthew et al. investigated the relationship between SIB with stress in the rhesus monkey model of self-injury (17). In a rat model, Yuan et al. illuminated the role of anxiety in vulnerability to self-injurious behavior (42). Here, we used mink as the animal model for the first time to investigate the genesis and development of SIB. We made several main behavioral observations and suggested that they are mechanistically and diagnostically indicative of SIB. SIB in minks arises spontaneously and is always accompanied by wounds, indicating the advantage of minks as the models of SIB. First, we confirmed that the frequency of self-biting and the repeating wheel is significantly increased in the minks spontaneously developing SIB. Meanwhile, the Results showed that the SIB group markedly reduced sleeping time, dietary amount, drinking frequency, defecation frequency, and body weight.

Furthermore, we investigated the pathological change of SIB in the mink brain. Our data showed that the microglial cells diffused hyperplasia in the brain parenchyma in minks with SIB but were not found in healthy minks. Meanwhile, activated Iba-1 microglial cells were increased in the brain parenchyma in the SIB group. In addition, we examined the levels of NfL and NfH, in the CSF and serum of mink with or without SIB. We observed increased levels of NfL and NfH in the CSF and serum of minks with SIB. In summary, these findings suggested that minks with SIB exhibited strong neurological illness signs.

Cyclic-AMP response element (CRE) binding protein (CREB) belongs to a large family of basic leucine zipper (bZIP)-containing transcription factors (43–45), which have long been known to be necessary for the formation of memories (46, 47). A recent study has shown that CREB signaling is dysfunctional in mice and humans with Alzheimer's disease (AD), a disease characterized by cognitive decline and memory impairments (25, 48). Illuminating the molecular mechanism of SIB is significant in developing new prevention and therapeutic strategies for SIB. However, most studies have focused on the environmental factors that reinforce SIB, but few have examined the underlying biological mechanisms of SIB. In our study, we observed that the phosphorylation levels of CREB were significantly increased in the brain tissues of minks with SIB, consistent with the previous study (29). Furthermore, we found for the first time that the CREB signaling is activated in the brain tissues of minks with SIB by detecting the mRNA levels of CREB target genes, including Bcl2, NOR1, FoxO4, and c-FOS.

Protein-protein interactions are essential for the transmission of signals from the extracellular space to the nucleus and for cell-cell communication. The transcriptional activity of p-CREB is further enhanced by the binding of CBP to phosphorylated CREB (p-CREB), which activates the transcription of CREB target genes (43).Given the significant roles of CREB in SIB development, we assumed that CBP was also associated with SIB development in minks. Indeed, CBP markedly up-regulated in the brain tissues of minks with SIB, indicating the significant role in the elicitation of SIB. Consistently, we also found that the expression was increased in the SIB mice and mink models induced by (±) Bay K 8644 agonist. And to our best knowledge, this is the first time to prove that CBP participates in SIB. However, the molecular mechanism that CBP expression increases in the brain tissues of minks with SIB still needs further investigation.

Small molecules that target protein-protein interactions are important research technologies for dissecting the biological functions of protein-protein interactions and potential therapeutics for many diseases (43). To validate the effect of CBP and CREB signaling on SIB development, we used a CBP-CREB interaction inhibitor to inhibit the interaction between CBP and CREB *in vivo*. Our results showed that sustained administration of CBP-CREB interaction inhibitor significantly reduced the expression of CREB target genes and relieved the neuronal damage in brain tissues of minks with SIB, which in turn gradually relieved the self-injury behavior and promoted the wound healing. Moreover, our findings shed light on the critical role of CBP in the genesis and development of SIB.

In conclusion, the study in this paper used a mink model of SIB to investigate the underlying mechanism involved in the genesis of SIB. Our results illustrated an induction of CBP and activation of CREB signaling, which can be regarded as a novel mechanism in the genesis of SIB. More importantly, the CBP-CREB interaction inhibitor markedly relieved the SIB severity *in vivo*, supplying an effective strategy for SIB therapy. These findings are also important supplements for the full understanding of SIB.

## Data availability statement

The original contributions presented in the study are included in the article/Supplementary material, further inquiries can be directed to the corresponding author/s.

## Ethics statement

This study was reviewed and approved by the Animal Administration and Ethics Committee of Qingdao Agricultural University, Animal Science and Technology College (Permit no. 2021-05-01). All animals involved in this manuscript were handled in strict accordance with good animal practice according to the Animal Ethics Procedures and Guidelines of the People's Republic of China.

## Author contributions

This study was designed by and GL. The manuscript was written by CL. The experiments were performed and analyzed by CL, XG, and HS. All authors read and approved the final manuscript.

## Funding

This study was funded by Shandong Modern Agricultural Technology & Industry System (SDAIT-21-01) and Technology Innovation Project of Chinese Academy of Agricultural Sciences grant CAAS-ASTIP-2016-ISAPS to GL. The funders play no role in study design, data collection and analysis, decision to publish or preparation of the manuscript.

## Conflict of interest

The authors declare that the research was conducted in the absence of any commercial or financial relationships that could be construed as a potential conflict of interest.

## Publisher's note

All claims expressed in this article are solely those of the authors and do not necessarily represent those of their affiliated organizations, or those of the publisher, the editors and the reviewers. Any product that may be evaluated in this article, or claim that may be made by its manufacturer, is not guaranteed or endorsed by the publisher.
