# Supplementary material for: A mink (Neovison vison) model of self-injury: Effects of CBP-CREB axis on neuronal damage and behavior
Source: Front Vet Sci. 2022 Nov 10;9:975112. doi: 10.3389/fvets.2022.975112 (PMC9686368; doi:10.3389/fvets.2022.975112)
Supplement: Supplementary file 1 [file Data_Sheet_1.docx]

Supplementary Material

# Supplementary Figures


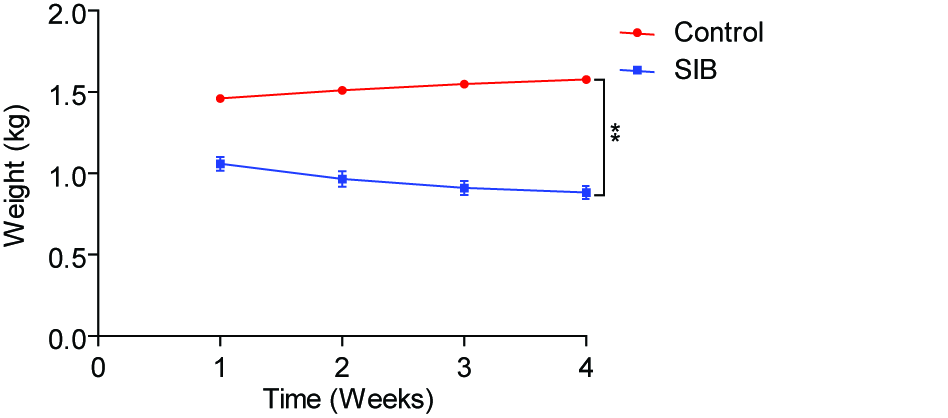


**Supplementary Figure 1. SIB reduced the body weight of minks.**  Scores for weight during 4 weeks (Data shown represent the mean ± SEM; ***p* < 0.01. Control, n = 10; SIB, n = 8).


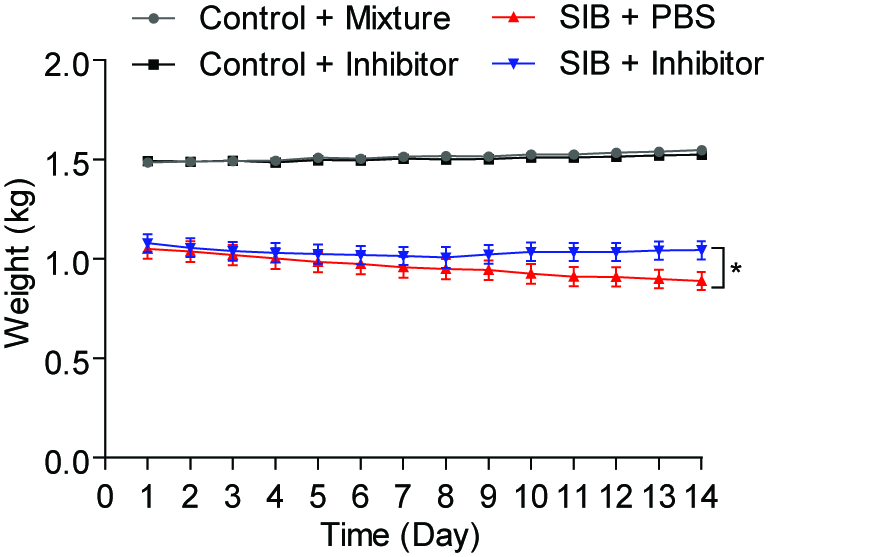


**Supplementary Figure 2. CBP-CREB interaction inhibitor significantly relieved the effect of SIB on the body weight of minks.** Scores for weight during 14 days treatment with inhibitor or PBS (Control + PBS, n = 10; Control + inhibitor, n = 10; SIB + PBS, n = 13; SIB + inhibitor, n = 14. Data shown represent the mean ± SEM; **p* < 0.05).

**Supplemental Figure 3. CBP-CREB interaction inhibitor markedly relieved the SIB.** Representative images of Mink (Control + PBS, n = 10; Control + Inhibitor, n = 10; SIB + PBS, n = 13; SIB + PBS, n = 14.).


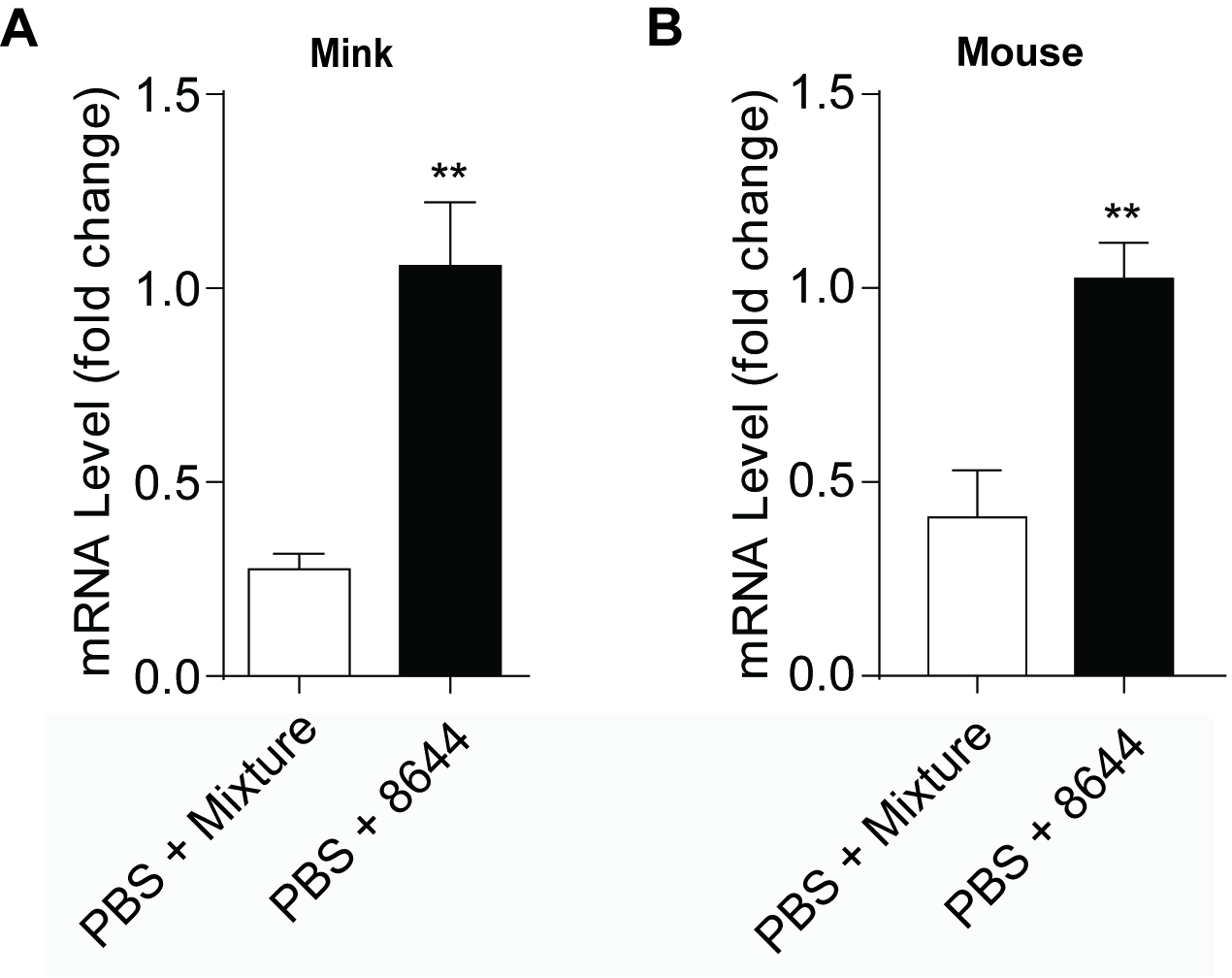


**Supplemental Figure 4. (±) Bay 8644 markedly upregulated the CBP levels in both mink and mouse models**.(A) The mRNA levels of CBP in mink brain tissues, assayed by qRT-PCR. (B) The mRNA levels of CBP in mouse brain tissues, assayed by qRT-PCR. Data shown represent the mean ± SEM, ***p* < 0.01.

**Supplemental Table**

**Supplemental Table 1. Group Information of Mice**

| Group | Description in detail |
| --- | --- |
| PBS + Mixture | Healthy mice injected with PBS, after 30 minutes were injected with Mixture (n = 10, both PBS and Mixture are at daily intervals.). |
| Inhibitor + Mixture | Healthy mice injected with Inhibitor, after 30 minutes were injected with Mixture (n = 10, both Inhibitor and Mixture are at daily intervals.). |
| PBS + 8644 | Healthy mice injected with PBS, after 30 minutes were injected with (±) Bay K 8644 (n = 10, both PBS and (±) Bay K 8644 are at daily intervals.). |
| Inhibitor + 8644 | Healthy mice injected with Inhibitor, after 30 minutes were injected with with (±) Bay K 8644 (n = 10, both Inhibitor and (±) Bay K 8644 are at daily intervals.). |

**Supplemental Table 2. Behavioral Categories**

| Behavioral Categories | Description in detail |
| --- | --- |
| Self-biting frequency | Record whenever any biting of the mink’s own body, then calculate the self-biting frequency. |
| Drinking frequency | Record whenever any consumption of water and then calculate the frequency. |
| Sleep | Record the amount of sleep. |
| Sleep position | Record the sleeping posture. |
| Repeating wheel | Running and flipping in circles, then calculate the frequency. |
| Food intake | Record the daily diet of minks. |
| Defecation frequency | Record the number of defecation, then calculate the defecation frequency. |

**Supplemental Table 3. Information on antibodies used for the correlation analysis.**

| Antibody | Source | Identifier |
| --- | --- | --- |
| p-CREB | Abcam | Cat#ab32096 |
| CBP | Santa Cruz | Cat#sc-7300 |
| p300 | Santa Cruz | Cat#sc-585 |
| Iba-1 | Abcam | Cat#EPR16589 |
| β-actin | Abcam | Cat#ab8226 |

**Supplemental Table 4. Enzyme-linked immunosorbent assay (ELISA) Kits Information.**

| Kit | Source | Identifier |
| --- | --- | --- |
| NfL | Cloud-Clone | Cat#42085596 |
| NfH | Cloud-Clone | Cat#42088738 |

**Supplemental Table 5. Sequences of primers for Real-time PCR analysis.**

| Species | Gene | Forward primer (5′- 3′) | Reverse primer (5′- 3′) |
| --- | --- | --- | --- |
| Mink | CBP | CCCTTCCCTACAGACATCAA | TGTGCCAACAGAACCAATC |
|  | P300 | CCAGCCTCAAACTACAAT | CAGCCATCACAGACAAAT |
|  | β-actin  c-Fos | GCGTGACATCAAGGAGAAGC  ATCACGACCAGCCAGGACC | AGCACCGTGTTGGCGTAG  ACAGTGGTGCCAATGGTCTT |
|  | BCL-2 | CCTACGGATTGACATTTCTG | GTGCTTCGCATTCTTGGA |
|  | NOR1 | GACTTCAGTGCCTTCGTG | CCTTACTCTGGTGGTCTTTC |
|  | FOXO4 | CCTGCTCACCTCCGATACAC | CGAAGTTGAAGTCCAGTCCC |
| Mouse | CBP | GCTGGCGGAGACCTTGACA | GCTGTATCAGTTTGGGTTT |
|  | β-actin | TGTGAACCACGAGAAGTATGA | AAGTCGCAGGAGACAACC |

**Supplemental Table 6. Numbers of mice that exhibited self-injurious behavior of each experimental day**

| Groups | n | Day 1 | Day 2 | Day 3 | Day 4 | Day 5 | Day 6 |
| --- | --- | --- | --- | --- | --- | --- | --- |
| PBS + Mixture | 0 | 0 | 0 | 0 | 0 | 0 | 0 |
| Inhibitor + Mixture | 0 | 0 | 0 | 0 | 0 | 0 | 0 |
| PBS + 8644 | 4 | 0 | 1 | 3 | 3 | 4 | 4 |
| Inhibitor + 8644 | 1 | 0 | 0 | 1 | 0 | 0 | 0 |

8644 represents the agonist (±) Bay K 8644, n represents the total number of mice that exhibited self-injurious behavior during the experiment.

**Supplemental Table 7. Numbers of minks that exhibited self-injurious behavior of each experimental day**

| Groups | n | Day 1 | Day 2 | Day 3 | Day 4 | Day 5 | Day 6 |
| --- | --- | --- | --- | --- | --- | --- | --- |
| PBS + Mixture | 0 | 0 | 0 | 0 | 0 | 0 | 0 |
| Inhibitor + Mixture | 0 | 0 | 0 | 0 | 0 | 0 | 0 |
| PBS + 8644 | 5 | 1 | 3 | 3 | 4 | 5 | 5 |
| Inhibitor + 8644 | 1 | 0 | 2 | 1 | 1 | 0 | 0 |

8644 represents the agonist (±) Bay K 8644, n represents the total number of minks that exhibited self-injurious behavior during the experiment.
